# 

**Published:** 1969-07

**Authors:** 


### *Contents*

| | Page |
|---|---|
| ANAPHYLACTOID PURPURA, M. E. R. Stoneman ... ... ... | 61 |
| MYELOID LEUKAEMOID REACTION IN A SURGICAL CASE, M. M. Ashraf ... ... ... ... ... ... ... ... | 72 |
| SIXTY YEARS OF STOKE PARK HOSPITAL, J. Jancar ... ... | 77 |
| SOUTH WEST ORTHOPAEDIC CLUB ... ... ... ... ... ... | 97 |
| OBITUARY, J. A. POCOCK ... ... ... ... ... ... | 100 |
| RECENTLY APPOINTED CONSULTANTS ... ... ... ... | 103 |

